# Dress

**Published:** 1887-10

**Authors:** 


### DRESS.

It is said that on one occasion Cogia Effendi, a Persian sage, dressed as a beggar, entered a house where a gay feast was being held. He was pushed hither and thither, hustled by one and another, and noticed kindly by no one. So Cogia withdrew, and repaired to his home, where he arrayed himself in his most splendid style, with jeweled shoes on his feet, a robe of cloth of gold on his back, and a turban glittering with a diamond aigrette on his head. Then, having hung at his side his saber, in the hilt of which flashed some valuable jewels, he returned to the feast. His entrance was the signal for attention on all sides. The guests, who before had rudely pushed him aside, now made way for his passing to and fro. The host came hastily toward him with the words, 'Welcome, my Lord Effendi, thrice welcome ; what will your lordship please to take ?' In reply, Cogia quaintly, but expressively stretched out his foot so that the jewel on his shoe sparkled ; and then, taking his golden robe in one hand, and holding it away from him, said, with bitter irony, 'Welcome, my lord coat, welcome, most excellent robe ; what will your lordship please to take ?—For,' said he, turning to his perplexed host, 'I ought to ask my coat what it will take, seeing that my welcome is due solely to it.

